# Overexpression of a *Fragaria vesca* NAM, ATAF, and CUC (NAC) Transcription Factor Gene (*FvNAC29*) Increases Salt and Cold Tolerance in *Arabidopsis thaliana*

**DOI:** 10.3390/ijms25074088

**Published:** 2024-04-06

**Authors:** Wenhui Li, Huiwen Li, Yangfan Wei, Jiaxin Han, Yu Wang, Xingguo Li, Lihua Zhang, Deguo Han

**Affiliations:** 1 Key Laboratory of Biology and Genetic Improvement of Horticultural Crops (Northeast Region), Ministry of Agriculture and Rural Affairs, National-Local Joint Engineering Research Center for Development and Utilization of Small Fruits in Cold Regions, College of Horticulture & Landscape Architecture, Northeast Agricultural University, Harbin 150030, China; wenhuili@neau.edu.cn (W.L.); xingguoli@neau.edu.cn (X.L.); 2 Horticulture Branch of Heilongjiang Academy of Agricultural Sciences, Harbin 150040, China; liuwanda1982@126.com

**Keywords:** *Fragaria vesca*, *FvNAC29*, high-salinity stress, low-temperature stress

## Abstract

The NAC (NAM, ATAF1/2, CUC2) family of transcription factors (TFs) is a vital transcription factor family of plants. It controls multiple parts of plant development, tissue formation, and abiotic stress response. We cloned the *FvNAC29* gene from *Fragaria vesca* (a diploid strawberry) for this research. There is a conserved NAM structural domain in the FvNAC29 protein. The highest homology between FvNAC29 and PaNAC1 was found by phylogenetic tree analysis. Subcellular localization revealed that FvNAC29 is localized onto the nucleus. Compared to other tissues, the expression level of *FvNAC29* was higher in young leaves and roots. In addition, *Arabidopsis* plants overexpressing *FvNAC29* had higher cold and high-salinity tolerance than the wild type (WT) and unloaded line with empty vector (UL). The proline and chlorophyll contents of transgenic *Arabidopsis* plants, along with the activities of the antioxidant enzymes like catalase (CAT), peroxidase (POD), and superoxide dismutase (SOD) under 200 mM NaCl treatment or −8 °C treatment, were higher than those activities of the control. Meanwhile, malondialdehyde (MDA) and the reactive oxygen species (ROS) content were higher in the WT and UL lines. *FvNAC29* improves transgenic plant resistance to cold and salt stress by regulating the expression levels of *AtRD29a*, *AtCCA1*, *AtP5CS1*, and *AtSnRK2.4*. It also improves the potential to tolerate cold stress by positively regulating the expression levels of *AtCBF1*, *AtCBF4*, *AtCOR15a*, and *AtCOR47*. These findings suggest that *FvNAC29* may be related to the processes and the molecular mechanisms of *F. vesca* response to high-salinity stress and LT stress, providing a comprehensive understanding of the NAC TFs.

## 1. Introduction

The environment in which plants grow is characterized by various stresses like drought, low temperature (LT), heavy metal excess, high salinity, and nutrient deficiency. This complex environment affects a wide range of plant physiological activities, including plant development and tissue formation, and has a significant negative impact on global horticultural crop yields [1,2,3,4]. Especially, high-salt and low-temperature environments can affect the ecological distribution, morphogenesis, and extensive physiological processes and biochemical reactions in plants. In agricultural production, the yield and quality of horticultural crops subjected to these two stresses are significantly reduced, to the extent that they threaten the food security of countries [5,6]. When plants are exposed to low temperatures, root function is impaired and stomatal closure is compromised while leaves wilt [7,8]. An increased cell membrane permeability of plant cells results in electrolyte imbalance. At the same time, LT causes the production of membrane lipid peroxides in cell membranes, leading to an increase in MDA and reactive oxygen species (ROS) [9]. High-salinity stress usually refers to the high-osmotic-potential environment caused by the ions chloride (Cl^−^) and sodium (Na^+^) in the soil [10]. When a plant is exposed to a high-salinity environment, its photosynthesis is affected, chloroplast synthesis is impeded while the plant is dwarfed, leaves are yellowed, and root length is inhibited [11]. Salt stress affects water uptake and produces ionotoxic effects, disrupts ion homeostasis, and leads to metabolic disorders, as well as contributes to an increase in ROS concentration [12,13]. Salt stress also severely affects potassium uptake by plants, and sodium can competitively inhibit potassium influx [14]. *DlNAC1* (*Dendranthema lavandulifolium*) was induced by low-temperatures [15]. The expressions of *NnNAC16*, *NnNAC25*, and *NnNAC70* from *Nelumbo nucifera* were increased under NaCl treatment [16]. *CaNAC035* expression is induced by low temperatures and salt [17]. Cold treatment conditions apparently increased the expression levels of 15 *KoNACs* by more than twofold [18]. *OsNAC14* was induced by LT and salt in leaves [19].

NAC TFs are one of the most important TF families in plants. And the c-terminal region is very changeable, whereas the n-terminal region is conserved [20,21,22]. NAC TFs were important TFs in regulating plant resistance to abiotic stresses like cold, heat, high salinity, and drought. NAC TFs were found to play roles in secondary wall formation [23,24], cell division [25], plant growth [26,27], and the responses of abiotic stresses. So far, much research has indicated that the NAC TFs are widely present in land plants [28,29,30,31]. Numerous studies have shown that NAC TFs effectively influence salt and LT tolerance in several plants. The expression of *GmNAC15* was responsive to salt treatments in the roots and leaves. Overexpression of *GmNAC15* could increase the soybean’ s resistance to salt [32]. *MdNAC047*, from *Malus hupehensis*, can regulate ethylene synthesis and may affect apple salt tolerance in this way [33].

Transcription factors are one of the regulatory genes, which act by regulating plant hormone signaling and gene expression [34]. Plant responses to NAC TFs are a vital part of the abiotic stress regulatory network. *MdNAC4* directly bound the promoter of the senescence-associated gene (SAG) *MdSAG39* and upregulated its expression [35]. *SNAC1* regulates the expression of genes containing NAC recognition sequences (NACRS) in the promoter region [36]. *SlNAC35*, *FtNAC31*, *AvNAC030*, *GmNAC2*, and *MbNAC25* may influence the tolerance of high-salinity stress of their respective transgenic plants by changing the scavenging capability of ROS [37,38,39,40,41].

In terms of the cold stress response, *CaNAC064* interacted with low-temperature-induced haplo-proteinase proteins, therefore improving resistance to the low-temperature stress [42]. *MaNAC1* regulates *Musa acuminata* cold stress response through interaction with *MaCBF1* and *MaICE1* genes [28]. Furthermore, it has also been shown that the *PbeNAC1* enhances cold tolerance in *Pyrus betulifolia* seedlings by interacting with DREB TFs (*PbeDREB1* and *PbeDREB2A*) [29]. *HuNAC20* and *HuNAC25* from *Hylocereus undatus* (Haw.) Britt. et Rose had a positive effect on increasing cold tolerance in transgenic *Arabidopsis*, which was achieved by promoting the stress-responsive gene expression (*RD29A*, *COR15A*, *COR47*, and *KIN1*) [43].

NAC TFs are abundantly present in *Fragaria vesca* L. Research on NAC TFs in *Fragaria vesca* demonstrates that NAC TFs are involved in strawberry growth, development, ripening, secondary cell wall formation, and flavonoid accumulation [44,45,46,47,48]. However, there are fewer studies related to the functionality of NAC TFs in response to LT stress in *F. vesca.*

A nuclear-localized NAC TF gene, *FvNAC29*, was isolated and cloned in our study. Experimental data indicated that LT and high salinity were two of the abiotic stresses that induce *FvNAC29*. Using *FvNAC29* transgenic *Arabidopsis thaliana* as the experimental material for stress treatment, it was determined that overexpression of *FvNAC29* enhances the high-salinity tolerance and cold tolerance of transgenic *Arabidopsis thaliana*. This research will provide a theoretical structure for further research into the functionality and molecular regulatory mechanisms of *FvNAC29*.

## 2. Results

### 2.1. Cloning and Bioinformatic Analysis of FvNAC29

**Figure 1:**
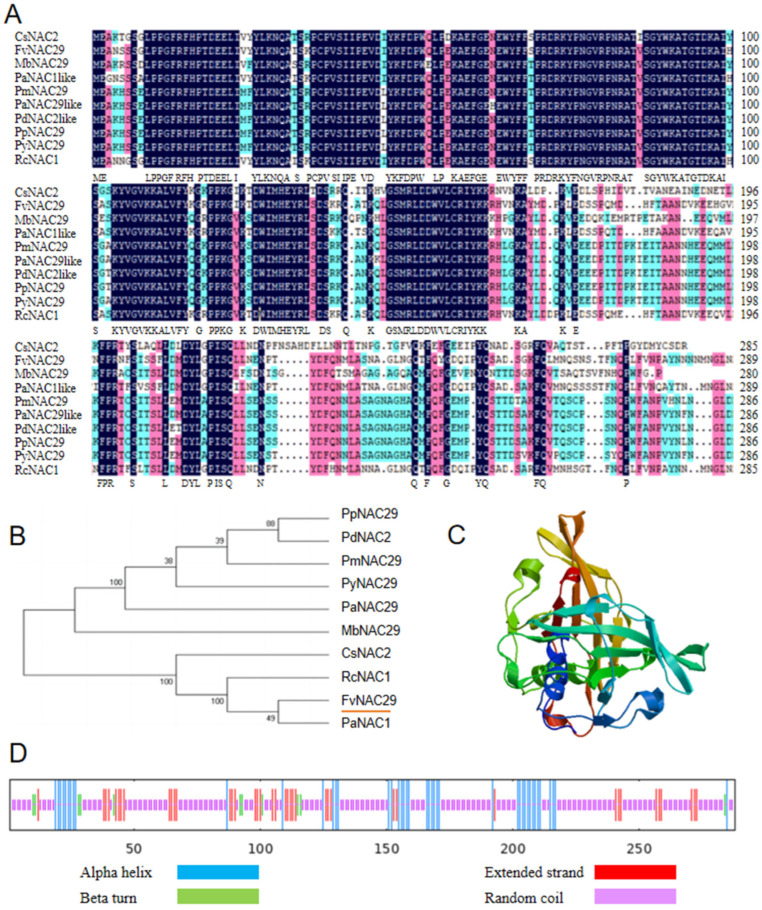
Contrast and evolutionary relationship between FvNAC29 and NAC transcription factors in different species, and prediction of FvMYB82 protein domains and structure. (**A**) Comparison between homology of FvNAC29 protein and NAC protein in other plants, with conserved amino acids shaded in different colors. The conserved regions of the amino acid sequence are marked by black and red boxes. (**B**) Phylogenetic tree analysis of NAC protein in *Fragaria vesca* and other plants. The red underline is the target protein. The accession numbers are as follows: CsNAC2 (*Cannabis sativa*, XP_030484994.1), MbNAC29 (*Malus baccata*, XP_050387456.1), PaNAC1-like (*Potentilla anserina*, XP_050372433.1), PmNAC29 (*Prunus mume*, XP_008221592.1), PaNAC29-like (*Prunus avium*, XP_021807478.1), PdNAC2-like (*Prunus dulcis*, XP_034200033.1), PpNAC29 (*Prunus persica*, XP_007223324.1), PyNAC29 (*Prunus yedoensis* var. *nudiflora*, PQM35312.1), RcNAC1 (*Rosa chinensis*, XP_024192624.1). (**C**) Tertiary structure of FvNAC029 protein predicted by Expasy. (**D**) Predicted protein secondary structure using the SPOMA.

The NAC transcription factor *FvNAC029* (XP_004297288.1) was isolated from *Fragaria vesca*, and the sequencing results are shown in Figure 1A. The full length of *FvNAC29* is 867 bp, and the ExPASy-ProtParam analysis predicted that the FvNAC29 protein consists of 288 amino acids, with the highest proportions of Asn (9.3%), Ser (8.0%), Lys (7.3%), and Pro (7.3%). FvNAC29 is a hydrophilic protein with a −0.744 grand average of hydropathicity (GRAVY).

Sequence analysis revealed that the FvNAC29 protein belongs to the NAM subfamily of the NAC TF family, has a NAM conserved domain, and has no obvious transmembrane structure. Our research constructed a phylogenetic tree to investigate the affinities of FvNAC29 by comparing the amino acid sequences of FvNAC29 protein with the sequences of NAC proteins from other species; FvNAC29 was determined to be similar to PaNAC1 (*Potentilla anserina*, XP_050372433.1), RcNAC1 (*Rosa chinensis*, XP_ 024192624.1), and CsNAC2 (*Cannabis sativa*, XP_030484994.1) with high homology (Figure 1B). The predicted secondary structure of the encoded proteins using SOPMA showed that the secondary structure of the FvNAC29 is composed up of 33.88% α-helix, 3.82% β turn, 67.1% random coil, and 14.24% extended strand (Figure 1C,D).

### 2.2. FvNAC29 Was Localizated onto Nucleus

**Figure 2:**
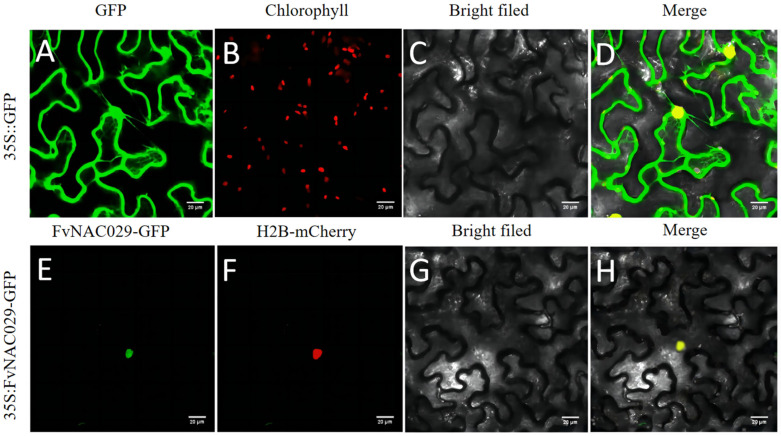
Subcellular localization of FvNAC29 in tobacco leaf epidermal cells. The 35S:GFP and 35S:FvMYB44-GFP plasmids were injected into the cells by *Agrobacterium tumefaciens* injection method. GFP protein was localized both in plasma membrane and in the nucleus. FvNAC029-GFP protein is located in the nuclei, overlapping with the nucleus marker H2B-mCherry, which is shown yellow. (**A**) GFP fluorescence. (**B**) Chlorophyll autofluorescence (red). (**C**,**G**) Bright-field images. (**D**,**H**) Merged. (**E**) FvNAC029-GFP. (**F**) mCherry (red). Bar = 50 μm.

To localize FvNAC29, a 35S::FvNAC29-GFP vector was constructed by fusing a GFP tag. After that, when the vector was transferred into the *Agrobacterium*, the outer epidermal cells of *Nicotiana benthamiana* leaves were injected with *Agrobacterium* for subcellular localization experiments. As shown in the figure (Figure 2), when control cells were observed with laser confocal microscopy, fluorescent signals were observed in both the cell membrane and nucleus (Figure 2), while 35S::FvNAC29-GFP protein fluorescence was observed only in the nucleus and co-localized with red signals from the nucleus marker H2B-mCherry. FvNAC29 was eventually determined to be present in the nucleus, based on the results of the subcellular localization described above.

### 2.3. Expression Analysis of FvNAC29

**Figure 3:**
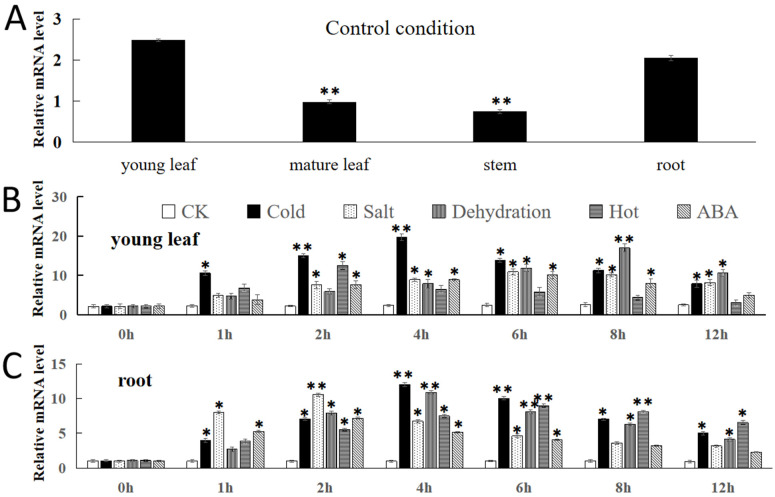
Expression pattern analysis of *FvNAC29* in *F. vesca* by quantitative qRT-PCR. (**A**) Expression of *FvNAC29* in different tissues in the non-stress environment. (**B**) Time-course of *FvNAC29* expression in young leaf in the control and under low-temperature (4 °C), salt (200 mM NaCl), dehydration (15% PEG6000), heat (30 °C), and abscisic acid (50 μM ABA) treatments. (**C**) Time-course of *FvNAC29* expression in root in the control and under low-temperature (4 °C), salt (200 mM NaCl), dehydration (15% PEG6000), heat (30 °C), and abscisic acid treatments (50 μM ABA). Error bars indicate the standard deviation. Asterisks above the error bars indicate a significant difference between the treatment and control (Student’s *t*-test; * *p* ≤ 0.05, ** *p* ≤ 0.01).

**Figure 4:**
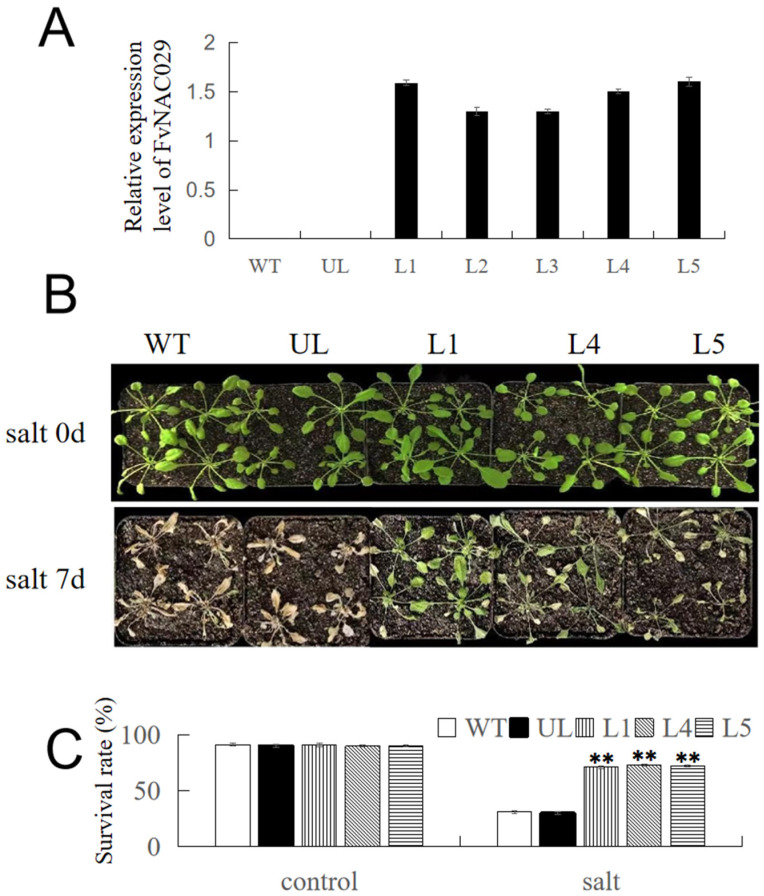
Growth of transgenic *A. thaliana* lines overexpressing *FvNAC29* under salt treatment. (**A**) Relative expression level of *FvNAC29* in WT, UL, and 5 *FvNAC29*-overexpression lines (L1, L2, L3, L4, and L5). (**B**) Phenotypes of the WT, UL, and transgenic lines (L1, L4, and L5) grown in the control environment, and salt treatment (irrigation with 200 mM NaCl for 7 days). Bar = 5 cm. (**C**) Survival percentages of WT, UL, and transformed lines (L1, L4, and L5) under the control environment and cold treatment. Asterisks indicate significant differences between WT and UL, and transformed lines (Student’s *t*-test, ** *p* ≤ 0.01).

For examining the tissue-specific expression of *FvNAC29* in different areas of *Fragaria vesca* seedlings, we analyzed the expression of *FvNAC29* in several of those tissues (young and mature leaves, stems, and roots) by qRT-PCR. Compared to stems and mature leaves, the expression level of *FvNAC29* was substantially higher in roots and the young leaves (Figure 3A). As Figure 4B,C show, *FvNAC29* was more sensitive to LT, salt, and dehydration stresses. Under four stresses, low-temperature stress, high-salinity stress, dehydration stress, and ABA stress, the expression of *FvNAC29* for young leaves exhibited the trend of growing and subsequently decreasing. The time at which the expression level of *FvNAC29* reached the maximum degree under each abiotic stress treatment was 4 h, 6 h, 8 h, and 6 h, in that order. In contrast, in the roots, the expression of *FvNAC29* showed a similar trend under each stress, with the expression reaching its highest point at 4 h, 2 h, 4 h, 6 h, and 2 h, in that order. The highest expression degree of *FvNAC29* was higher than those of other treatments when under LT, dehydration, and salt stresses in both organs (Figure 3B,C). From the above results, *FvNAC29* was significantly induced by LT, salt, and dehydration stresses.

### 2.4. Overexpression of FvNAC29 Increases Tolerance to Salt Stress

For revealing the role of *FvNAC29* on salt and LT tolerance in *Arabidopsis*, we constructed transgenic *Arabidopsis* lines (L1 to L5, Figure 4A) overexpressing *FvNAC29*. Among them, L1, L4, and L5 lines showed a higher expression of *FvNAC29* (Figure 4A), and these three lines were selected for following abiotic stress treatments. In the absence of any stress treatment, the development of each strain was essentially the same. WT, UL, L1, L4, and L5 were watered using a NaCl solution of 200 mM for seven days, and the irrigation fluid was changed into water after seven days. Phenotypic observations showed that all plants showed some degree of wilting, yellowing, and growth retardation under NaCl irrigation conditions, the leaves of L1, L4, and L5 were still green, and WT and UL plants were severely affected, showing dehydration and wilting. After stopping the 200 mM NaCl irrigation treatment and rinsing the plants with water for 3 days, WT and UL plants showed extensive wilting, whereas the three lines overexpressing *FvNAC29* (L1, L4, and L5) were not further affected and had higher survival rates of 68%, 73%, and 71%, respectively (Figure 4B,C). The results indicated that overexpression of *FvNAC29* in *Arabidopsis*, to a certain extent, could alleviate the damage caused by high-salinity stress.

**Figure 5:**
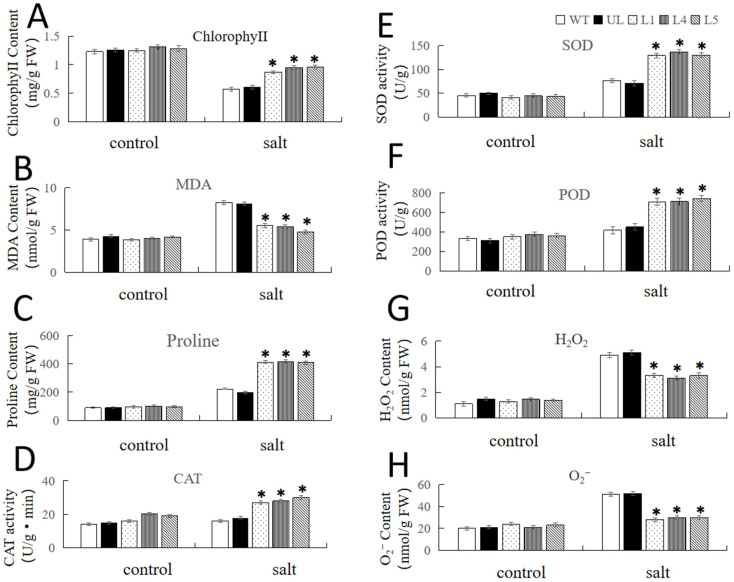
Physiological indicators in transgenic *A. thaliana* lines overexpressing *FvNAC29* under salt treatment. Contents of (**A**) chlorophyll, (**B**) MDA, (**C**) proline, (**G**) H_2_O_2_, and (**H**) O_2_^−^ and the activities of (**D**) CAT, (**E**) SOD, and (**F**) POD in the WT, UL, and *FvNAC29*-OE lines (L1, L4, and L5) under 200 mM NaCl treatment for 7 days. Significant differences are marked with asterisks above the error bar (Student’s *t*-test, * *p* ≤ 0.05). The levels of indicators in the WT were used as the control.

We examined the physiological indices related to stress tolerance (chlorophyll, proline, SOD, POD, CAT) in each strain, and the indices were basically at the same level between the transgenic strain and the WT and UL strains before the stress treatment. When the transgenic lines (L1, L4, L5) were treated with high salinity, the chlorophyll and proline contents and the activities of a portion of the antioxidant enzymes (CAT, SOD, POD) were much higher than those of the WT and UL lines. Compared to the WT and UL lines, the MDA, H_2_O_2_, and O_2_^−^ contents, which often represent the degree of plant damage under abiotic stresses, were lower in *Arabidopsis* lines overexpressing *FvNAC29* (Figure 5).

**Figure 6:**
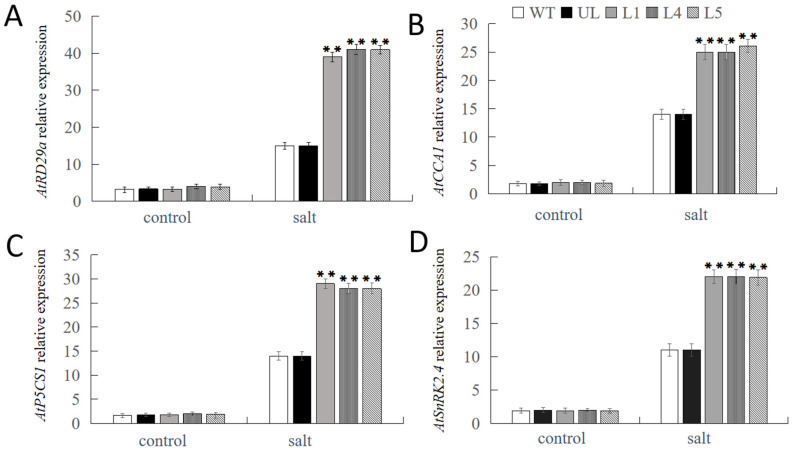
Expression levels of salt-related genes in WT, UL, and transformed *A. thaliana* overexpressing *FvNAC29* under salt treatment. Relative expression levels of (**A**) *AtRD29a*, (**B**) *AtCCA1*, (**C**) *AtP5CS1*, and (**D**) *AtSnRK2.4* in the WT, UL, and *FvNAC29*-OE lines (L1, L4, and L5). Data are the average of three replicates. Significant differences are marked with an asterisk above the error bar (Student’s *t*-test, ** *p* ≤ 0.01).

We know from the aforementioned experiments that *FvNAC29* can significantly increase the transgenic plants’ high salt tolerance. We investigated the expression levels of four genes linked to salt stress in the plants of each treatment, *AtRD29a*, *AtCCA1*, *AtP5CS1*, and *AtSnRK2.4*, in order to uncover the molecular mechanism regulating it (Figure 6). The expression levels of these four genes were found to be significantly higher in L1, L4, and L5 transgenic lines under salt treatment than in WT and UL lines. This suggests that *FvNAC29* could increase transgenic plant tolerance to high salt stress by influencing downstream stress-related gene expression.

### 2.5. Overexpression of FvNAC29 Increases Tolerance to Cold Stress

**Figure 7:**
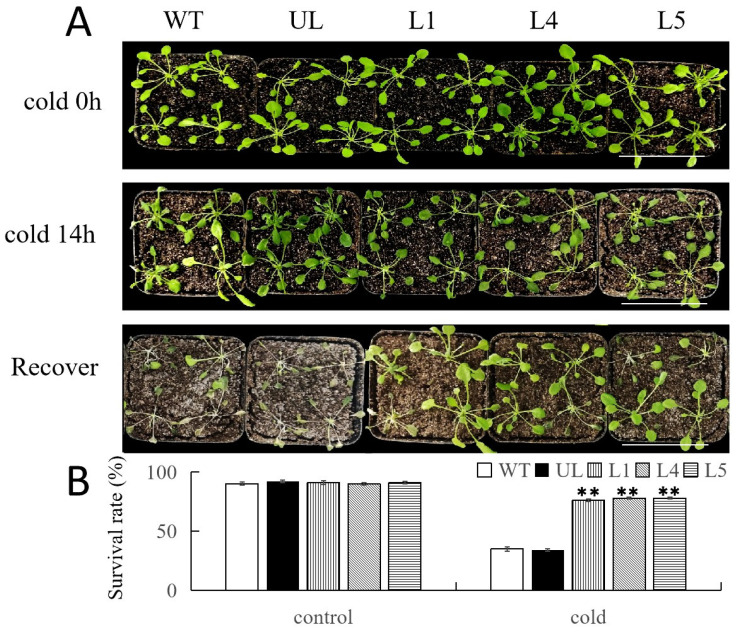
Growth of transgenic *A. thaliana* overexpressing *FvNAC29* under low-temperature treatment. (**A**) Phenotypes of WT, transformants with empty vector (UL), and *FvNAC29*-overexpressing lines (L1, L4, and L5) under the control environment (22 °C), cold treatment (−8 °C for 7 h), and after recovery. Bar = 5 cm. (**B**) Survival rate of WT, UL, and transgenic lines under the control environment and cold treatment. Three replicates were performed. Asterisks indicate a significant difference between the different lines (Student’s *t*-test, ** *p* ≤ 0.01).

Similarly, in the absence of any abiotic stress treatment, all *Arabidopsis* lines grew almost identically. When these lines were subjected to 14 h of low-temperature treatment at −8 °C, the leaves of WT and UL lines exhibited slight water loss, but the growth of the three overexpression lines (L1, L4, and L5) did not change significantly at this time. After one week of recovery at room temperature (24 °C), the *Arabidopsis* lines overexpressing *FvNAC29* maintained healthy growth. In contrast, WT and UL lines were heavily affected by cold stress, with yellowing and wilting of the leaves. The survival rates of L1, L4, and L5, after stress treatment, were 77%, 80%, and 80%, respectively, while those of the WT lines and UL lines were only 32% and 35% (Figure 7A,B), respectively.

**Figure 8:**
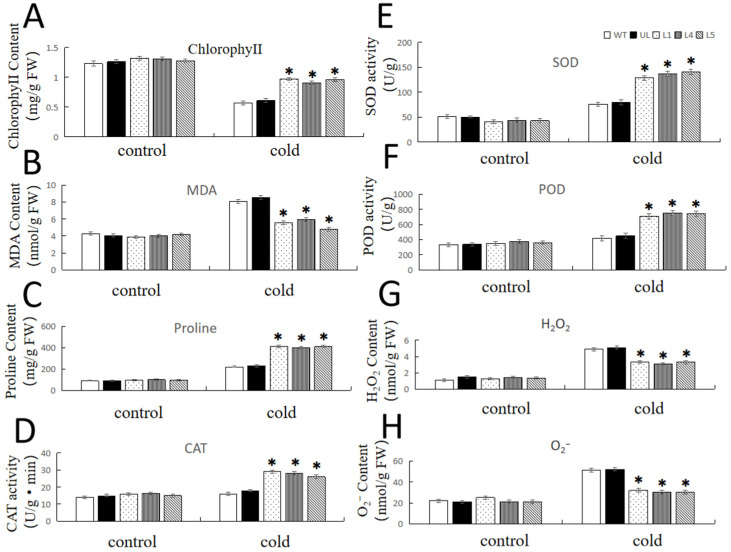
Physiological indicators in transgenic *A. thaliana* lines overexpressing *FvNAC29* under low-temperature treatment. Contents of (**A**) chlorophyll, (**B**) MDA, (**C**) proline, (**G**) H_2_O_2_, and (**H**) O_2_^−^ and the activities of (**D**) CAT, (**E**) SOD, and (**F**) POD in the WT, UL, and *FvNAC29*-overexpressing lines (L1, L3, and L4) under the non-stress environment (22 °C) or cold treatment (−8 °C for 14 h). Asterisks above each error bar indicate obviously significant differences between transgenic lines (L1, L4, and L5), UL, and the WT (Student’s *t*-test, * *p* ≤ 0.05). The levels of indicators in the WT were used as the control.

The MDA, H_2_O_2_, and O_2_^−^ contents of the WT and UL lines increased considerably after cold stress, whereas these three parameters increased only slightly in transgenic *Arabidopsis*. The chlorophyll and the proline contents with the activities of CAT, SOD, and POD were higher in the lines overexpressing *FvNAC29* than those in the WT and UL lines (Figure 8). These data reveal that overexpression of *FvNAC29* in *Arabidopsis* can raise the activities of antioxidant enzymes while lowering the degree of membrane lipid peroxidation, thus enhancing the low-temperature tolerance of *Arabidopsis*.

**Figure 9:**
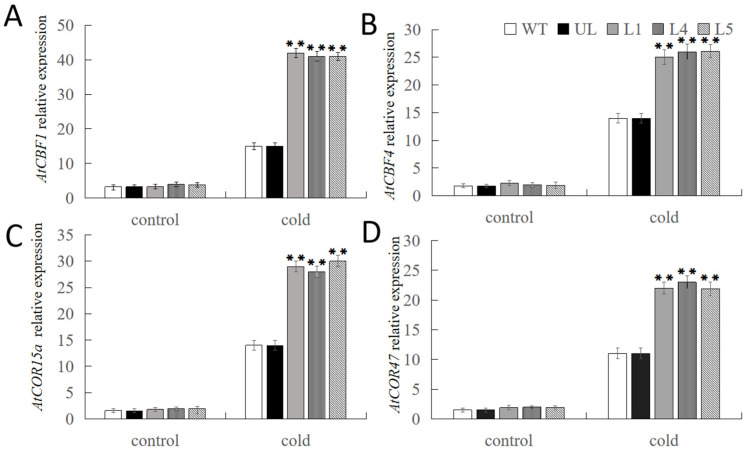
Expression of chilling-related genes in transgenic *A. thaliana* lines overexpressing *FvNAC29* under low-temperature treatment. Relative expression levels of (**A**) *AtCBF1*, (**B**) *AtCBF4*, (**C**) *AtCOR15a*, and (**D**) *AtCOR47* in the WT, UL, and transgenic lines (L1, L4, and L5). Data are the average of three repetitions. Asterisks indicate extremely significant differences between the transgenic line and the WT (Student’s *t*-test, ** *p* ≤ 0.01).

Comparably, we looked at the expression levels of genes linked to low-temperature stress in each treatment, such as *AtCBF1*, *AtCBF4*, *AtCOR15a*, and *AtCOR47*, in order to investigate the molecular role of *FvNAC29* in the control of cold stress response in plants. Lines overexpressing *FvNAC29* showed significantly higher expression levels of low-temperature-responsive-related genes under high-salt treatment compared to control groups (WT and UL, Figure 9). This suggests that *FvNAC29* can enhance transgenic plant tolerance to low-temperature stress by influencing the expression of downstream stress-related genes.

## 3. Discussion

Understanding how NAC transcription factors respond to external stresses is beneficial for horticultural crop research and breeding. NAC TFs are essential for controlling how plants grow, develop, and react to adverse environments. However, particularly for fruit crops, the mechanism behind the NAC TF-mediated stress response is not entirely understood.

In our research, we cloned the *FvNAC29* gene from *Fragaria vesca*. FvNAC29 has a NAM subfamily-like domain. The *FvNAC29* gene was isolated and cloned from *F. vesca*. Protein sequence analysis showed that FvNAC29 has a highly conserved n-terminus, similar to other NAC TFs. Analysis of the structural domains determined that FvNAC29 contains a typical NAM subfamily-like structural domain, demonstrating that it is a typical NAC TF.

There are many studies on the subcellular localization of the NAC TF. It was determined that the fluorescent signal could only be observed in the nucleus. Our results of subcellular localization are consistent with the results of MaNAC154 (*Musa acuminata*), CarNAC4 (*Cicer arietinum* L.), and OsNAC083 (*Oryza sativa*) [49,50,51]. While the majority of NAC proteins are found in the nucleus, some are only found in the intracellular membrane and are referred to as NAC membrane-bound TFs (NTLs) [52]. Among *Arabidopsis* NTLs, some such as AtNTL4 and AtNTL6 exist in the plasma membrane, whereas some such as AtNTL1 and ANAC089 were found to exist in the endoplasmic reticulum [53,54,55,56,57].

Gene expression is cell- or tissue-specific throughout plant growth and is usually manifested as varying gene expression levels in various tissues or organs. Therefore, to verify our speculation, we analyzed the expression of *FvNAC29*. Figure 3 demonstrates that the expression level of the *FvNAC29* in *Fragaria* displayed tissue specificity. The expression of *FvNAC29* in the stems and mature leaves was much lower than those in young leaves or roots in the same plant at the same time. This may indicate that *FvNAC29* is more sensitive to abiotic stresses in newborn organs. NACs exist in various plants, and a similar tissue-specific expression is also present in plants such as poplar (*Populus euphratica*), hardy Rubber Tree (*Eucommia ulmoides*), and Wintersweet (*Chimonanthus praecox*) [58,59,60]. Different stress-induced changes in *FvNAC29* expression differed under abiotic stresses, and *FvNAC29* was more sensitive to LT, high-salinity, and dehydration stress, and more in-depth studies can be conducted in the future to investigate the mechanism by which *FvNAC29* responds to abiotic stress, specifically dehydration stress.

External stress causes plants to release a lot of ROS quickly. And it can upset the intracellular redox balance while severely damaging cells [61]. The primary components of the plant protective enzyme system are CAT, SOD, and POD, which may remove ROS from cells in tough growth environments to lessen the harm that ROS causes to cells [62]. Their activity can therefore reflect the degree of plant damage in unfavorable environments. Plants overexpressing *MbNAC25* raise the activity of antioxidant enzymes CAT, SOD, and POD [41]. The changes to the contents of MDA, chlorophyll, and proline are frequently utilized to illustrate the strength of plant stress tolerance [63,64,65]. MusaNAC042 from *Musa acuminata* leads to transgenic bananas with higher proline and lower MDA contents under high-salinity treatment with 250 mM NaCl [66]. The chlorophyll contents of the transgenic *Arabidopsis*-overexpressed TaNAC67 from *Triticum aestivum* L. were higher than the contents of the control plants under salt treatment [67]. After three days of cold stress at 4 °C, the MDA content of *CaNAC064*-silenced chili peppers was increased. Overexpression of *CaNAC064* effectively increased cold tolerance in *A. thaliana* [42].

Drought and salt stress are among the many stress reactions that ABA mediates [68]. NAC TFs frequently control plant salt tolerance via this route. Through the control of seven genes, including *OsEREBP2* and *OsSIK1*, rice *OsNAC45* may have a regulatory function in the ABA response and salt tolerance [69]. *IbNAC3* from *Ipomoea batatas* influences *A. thaliana*’s resistance to salt and drought stress by interacting with two ABA-related genes, *ERA1* and *MPEG57* [70]. By binding to the promoters of the ABA-metabolism-related genes *AtABA1* and *AtAAO3*, *SlNAC4* increases the expression of these genes and improves *Arabidopsis*’s resistance to salt and drought stress [71]. It was discovered that ABA stimulated the expression of many genes, such as *ANAC096* [72], *GmNAC06* [73], and *OsNAC52* [74], which positively control salt tolerance in plants. This article focuses on four ABA-pathway-related genes: *AtRD29a* [75], *AtCCA1* [76], *AtP5CS1* [77], and *AtSnRK2.4* [78]. *FvNAC29* improved salt tolerance in *Arabidopsis* by upregulating the expression of these four genes.

One important mechanism in plants’ reaction to cold stress is the CBF pathway. TFs like MYB15 and ICE1 regulate the expression of CBF genes. By interacting with CRT/DRE cis-acting sites on COR promoters, CBFs can also regulate COR genes [79,80]. Previous research reports have demonstrated the involvement of *CBF1* and *CBF4* in the cold induction process of *COR15a*. Whereas *CBF4* is involved in ABA-dependent signaling [81], CBF4 transcripts are also induced by cooling and LT, demonstrating that *CBF4* acts in both drought and low-temperature stresses [82]. *CBF4* binds to *COR47*, a typical CBF downstream gene linked to cold regulation in *Arabidopsis*, and increases the expression of *COR47*, which contains a DRE cis-acting element [83]. Numerous studies have shown how NAC TFs function in a CBF-dependent way in response to cold stress. *MdCBF1* and *MdCBF3* promoters can be directly bound by MdNAC104 for regulating the apple’s response to cold stress [84]. *MaNAC1* participates in the ICE1-CBF cold signaling pathway by acting as a downstream gene of *MaICE1* and interacting with *MaCBF1* [28]. *SlCOR518* and *SlCOR413IM1* were highly expressed in *SlNAC35*-overexpressing plants at low temperatures [37]. Via the DREB/CBF-COR pathway, *LlDREB1* can bind to the promoter of *LlNAC2* and play a role of plant abiotic stress [85].

**Figure 10:**
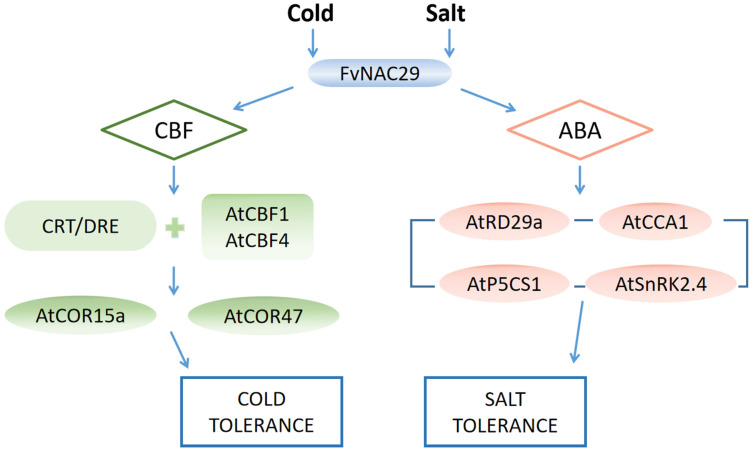
A working model for the involvement of *FvNAC29* in the regulation of cold and salt stress in plants. *FvNAC29* receives signals to be activated when plants are exposed to low-temperature or high-salt environments. On the one hand, *FvNAC29* participates in the CBF pathway, binds to the promoters of *AtCBF1* and *AtCBF4* to promote their binding to CRT/DRE, and activates the expression of the downstream genes, *AtCOR15a* and *AtCOR47*, to enhance the plant’s tolerance to the low-temperature environment. On the other hand, *FvNAC29* participates in the ABA signaling pathway and promotes the expression of the downstream genes *AtRD29a*, *AtCCA1*, *AtP5CS1*, and *AtSnRK2.4* to realize the enhancement in salt tolerance in plants.

In this research, proline content and CAT, SOD, and POD activities were increased in *FvNAC29* transgenic *Arabidopsis* after salt and cold treatments. The plants overexpressed FvNAC29 revealed less rise in MDA, O_2_, and hydrogen peroxide contents and more stable changes in chlorophyll content than WT and UL plants after stress (Figure 5 and Figure 8). These findings suggest that high-salinity stress and cold stress can induce *FvNAC29* to engage the abiotic stress response mechanisms in land plants. In addition, overexpression of *FvNAC29* increased plant resistance to stresses in many ways, including by affecting plant antioxidant activity and enhancing transgenic plant tolerance to low-temperature stress by influencing the expression of downstream stress-related genes (Figure 10). In this study, the function of *FvNAC29* was heterologously analyzed in *Arabidopsis thaliana*, and whether *FvNAC29* can perform a similar function in *Fragaria vesca* ontogeny needs to be further investigated. This study demonstrated that *FvNAC29* can effectively affect the cold tolerance of *A. thaliana*, which can provide some reference value for the production of cultivated strawberry in cold regions.

## 4. Materials and Methods

### 4.1. Plant Materials

Diploid wild-type forest strawberry, which is a model plant for the study of octoploid cultivated strawberries (*Fragaria × ananassa*) and other *Rosaceae* crops, was used as the experimental material for this experiment [86]. The *F. vesca* seedlings were sown in a mixed substrate in a ratio of 2:1 of the nutrition soil to vermiculite and cultivated in a constant-22 °C incubator where the photoperiod was set to 16 h light and 8 h darkness (with 70% relative humidity) [87].

### 4.2. Expression Patterns of FvNAC29

For functional analysis of *F. vesca*, seedlings from the same batch were grouped and subjected to separate stress treatments after two weeks of growth. Stress treatment was carried out with reference to previous studies [88]. The first group without any stress treatment was used as a control group in a constant-temperature incubator. The remaining five groups were treated with 4 °C, 37 °C, 15% PEG6000, 200 mM NaCl, and 100 μM ABA in order, which were used to simulate the plants under LT, heat, drought, high-salinity, and ABA stress environments. At each time point shown in Figure 4, different parts were sampled, and after sampling, they were frozen in liquid nitrogen (N_2_). Then, they were kept in the refrigerator (set to −80 °C) for the RNA isolation and cloning of *FvNAC29* [41].

### 4.3. RNA Extraction and Cloning of FvNAC29

Young and mature leaves, stems, and roots from the incubator-cultivated seedlings of *F. vesca* were selected as experimental materials. We used these materials to isolate total RNA, which was later purified, using the Universal Plant Total RNA Isolation Kit from Vazyme (Nanjing, China) [88]. The first-strand cDNA was reverse-transcribed, using the RNA as the template. In addition, a pair of specific primers was designed (*FvNAC29*-F/R; Table S1). Then, we ligated the PCR product to the ASY-T1 vector (TransGen Biotech, Beijing, China) and subsequently sent them for sequencing.

### 4.4. Subcellular Localization Analysis of FvNAC29

We utilized a pair of primers with *SalI* and *BamHI* enzyme digestion sites (*FvNAC29*-*slF*/*slR*; Table S1) to amplify the CDS of *FvNAC29*. The FvNAC29-GFP transient expression vector was constructed by double-digesting of the PCR product and pCAMBIA1300 vector using two restriction endonucleases, *SalI* and *BamHI*. We transformed the constructed transient expression vector pCAMBIA1300-FvNAC29-GFP into the *Agrobacterium* strain GV3101, and used the same process to transform the 35s:H2B-mCherry and empty 35S::GFP as well. Then, the successfully transformed *Agrobacterium* sap was injected into outer epidermal cells from *Nicotiana benthamiana* leaves by the *Agrobacterium* injection method. Plants were cultured at 24 °C for 3 days and then the fluorescent signal was photographed using a confocal microscope (Zeiss AxioImager D2, Zeiss, Oberkochen, Germany) [89].

### 4.5. Sequence Analysis and Structure Prediction of FvNAC29

We obtained the primary structure of the FvNAC29 protein by ExPASy-ProtParam with ExPASy-ProtScale (http://www.expasy.org/, accessed on 5 December 2022) analysis website. Amino acid sequences of FvNAC29 protein and the NAC proteins of other plants were collected from NCBI (https://www.ncbi.nlm.nih.gov/, accessed on 5 December 2022). A phylogenetic genetic tree was constructed on MEGA7 [90]. And the multiple sequence comparison was performed by DNAMAN 9.0. The structural domains of FvNAC29 protein were predicted in the SMART (http://smart.embl-heidelberg.de/, accessed on 7 December 2022) and InterPro (https://www.ebi.ac.uk/interpro/, accessed on 7 December 2022) databases. SPOMA was used to predict the secondary structure of FvNAC29 protein (http://npsa-pbil.ibcp.fr/cgi-bin/npsa_automat.pl?page=npsa_sopma.html/, accessed on 11 December 2022). The SWISS-MODEL was used to predict its tertiary structure (https://swissmodel.expasy.org/, accessed on 11 December 2022) [91].

### 4.6. Expression Analysis of FvNAC29

The expression analysis of *FvNAC29* was utilized to evaluate the degree of *FvNAC29* in several organs under multiple stresses. The qPCR primers (*FvNAC29*-qF/qR; Table S1) were designed. In addition, the reaction system of qPCR was based on the method of Li et al. [88]. For the internal reference gene, we finally used the *FvActin* (XM_011471474.1, *F. vesca*), and the corresponding primer was designed (*FvActin*-F/R; Table S1). The 2^−∆∆Ct^ method was utilized to estimate the expression of target genes [92].

### 4.7. Generation of A. thaliana Lines Overexpressing FvNAC29

The *FvNAC29*-OE vector was constructed by amplifying the 5′ end (*SalI* restriction enzyme digestion site) and 3′ end (*BamHI* site) of the *FvNAC29* cDNA with the *FvNAC29*-F and *FvNAC29*-R primer pairs to ligate it into the pCAMBIA1300 vector. The *FvNAC29* overexpression vector was transferred into the *Agrobacterium* GV3101 to transform *A. thaliana* (Col-0), using the inflorescence-mediated method. And after that, positive transgenic lines were transferred to MS solid screening medium, which was supplemented with 50 mg/L kanamycin [93]. We used qRT-PCR to identify the *FvNAC29* overexpression lines. The screened T3 generation lines were used for further analysis, and controls of WT and UL were set up.

### 4.8. Stress Treatment

As experimental materials, WT, UL, and L1, L4, and L5 transgenic lines in tissue culture were used. Seedlings were transplanted into pots containing the mixed substrate (soil/vermiculite = 2:1) after exposure of cotyledons. We chose the seedlings with stable growth conditions, and then divided them into two groups with one group containing 20 seedlings. And every four plants were planted in the same pot. One group was used for salt stress treatment and the other group was used for cold stress treatment. For the high-salt treatment group, we irrigated them with NaCl solution at 200 mM every two days, and after seven days, the seedlings were irrigated with fresh water to remove the residual NaCl solution [94]. For the low-temperature-treated group, seedlings were exposed to −8 °C for 14 h and then moved to normal-room-temperature (24 °C) conditions for one week.

### 4.9. Determination of Physiological Indexes

Survival and physiological parameters of *Arabidopsis* lines (WT, UL, L1, L4, L5) were subsequently measured. The absorbance of chlorophyll solution was determined by the method of Han and Ren [95,96], and the formula for calculating the content of chlorophyll was referred to the method of Zhang [97]. Antioxidant enzyme (CAT, SOD, POD) activities were determined by the method of Zhang [97]. H_2_O_2_ and O_2_^−^ contents were assayed by diaminobenzidine (DAB) and nitro blue tetrazolium (NBT), respectively [98]. The MDA content in the samples was determined by TBA [99]. And the proline content was determined based on the sulfosalicylic acid method [99].

### 4.10. Expression Analysis of Salt- and Cold-Stress-Related Response Genes

Using *FvActin* as an internal reference gene, qPCR analysis was performed on WT, UL, L1, L4, and L5 to investigate the expression levels of response genes related to abiotic stress. Refer to the *FvNAC29* qPCR system above for the reaction system. The 2^−∆∆Ct^ method was utilized to estimate the expression of target genes [100].

### 4.11. Statistical Analysis

All data of our research were collected from three technical replicates. The mean values of the replicated trials were used as values for the corresponding samples. Significance analyses were performed using SPSS 21 (IBM, Chicago, IL, USA), and Student’ s *t*-test was performed with one-way ANOVA [101]. Significance is expressed as Pearson (* *p* ≤ 0.05; ** *p* ≤ 0.01) correlation coefficient.

## 5. Conclusions

In this research, we cloned the nuclear-localized NAC TF gene *FvNAC29* from *F. vesca* and investigated its expression level and regulatory mechanism under normal and abiotic stress conditions. *FvNAC29* is highly sensitive to salt and cold stresses. *FvNAC29* may affect the stress response of plants by regulating their antioxidant activity and regulating the downstream expression of resistance-related genes. Taken together, *FvNAC29* may be regulating plant tolerance under abiotic stress.

## Data Availability

Data are contained within the article and Supplementary Materials.

## Supplementary Materials

The following supporting information can be downloaded at https://www.mdpi.com/article/10.3390/ijms25074088/s1.

## References

[1] Han D., Zhou Z., Du M., Li T., Wu X., Yu J., Zhang P., Yang G. (2020). Overexpression of a *Malus xiaojinensis* WRKY transcription factor gene (*MxWRKY55*) increased iron and high salinity stress tolerance in *Arabidopsis thaliana*. In Vitro Cell. Dev. Biol. Plant.

[2] Zandalinas S., Fritschi F., Mittler R. (2021). Global warming, climate change, and environmental pollution: Recipe for a multifactorial stress combination disaster. Trends Plant Sci..

[3] Han D., Yang G., Xu K., Shao Q., Yu Z., Wang B., Ge Q., Yu Y. (2013). Overexpression of a *Malus xiaojinensis Nas1* gene influences flower development and tolerance to iron stress in transgenic tobacco. Plant Mol. Biol. Rep..

[4] Han D., Shi Y., Yu Z., Liu W., Lv B., Wang B., Yang G. (2015). Isolation and functional analysis of *MdCS1*: A gene encoding a citrate synthase in *Malus domestica* (L.) Borkh. Plant Growth Regul..

[5] Thakur P., Nayyar H. (2012). Facing the cold stress by plants in the changing environment: Sensing, signaling, and defending mechanisms. Plant Acclimation to Environmental Stress.

[6] Farooq M., Hussain M., Wakeel A., Siddique K.H. (2015). Salt stress in maize: Effects, resistance mechanisms, and management. A review. Agron. Sustain. Dev..

[7] Guo H., Wu T., Li S., He Q., Yang Z., Zhang W., Gan Y., Sun P., Xiang G., Zhang H. (2019). The methylation patterns and transcriptional responses to chilling stress at the seedling stage in rice. Int. J. Mol. Sci..

[8] Theocharis A., Clément C., Barka E.A. (2012). Physiological and molecular changes in plants grown at low temperatures. Planta.

[9] Singh A., Mehta S., Yadav S., Nagar G., Ghosh R., Roy A., Chakraborty A., Singh I.K. (2022). How to Cope with the Challenges of Environmental Stresses in the Era of Global Climate Change: An Update on ROS Stave off in Plants. Int. J. Mol. Sci..

[10] Ismail A., Takeda S., Nick P. (2014). Life and death under salt stress: Same players, different timing?. J. Exp. Bot..

[11] Julkowska M., Testerink C. (2015). Tuning plant signaling and growth to survive salt. Trends Plant Sci..

[12] Wang Z., Wang M., Liu L., Meng F. (2013). Physiological and proteomic responses of diploid and tetraploid black locust (*Robinia pseudoacacia* L.) subjected to salt stress. Int. J. Mol. Sci..

[13] Bazihizina N., Colmer T., Cuin T., Mancuso S., Shabala S. (2019). Friend or foe? Chloride patterning in halophytes. Trends Plant Sci..

[14] Mulet J., Porcel R., Yenush L. (2023). Modulation of potassium transport to increase abiotic stress tolerance in plants. J. Exp. Bot..

[15] Yang Y., Zhu K., Wu J., Liu L., Sun G., He Y., Chen F., Yu D. (2016). Identification and characterization of a novel NAC-like gene in chrysanthemum (*Dendranthema lavandulifolium*). Plant Cell Rep..

[16] Zhao S., Jiang T., Zhang Y., Zhang K., Feng K., Wu P., Li L. (2022). Identification of the NAC Transcription Factors and Their Function in ABA and Salinity Response in *Nelumbo nucifera*. Int. J. Mol. Sci..

[17] Zhang H., Ma F., Wang X., Liu S., Saeed U., Hou X., Zhang Y., Luo D., Meng Y., Zhang W. (2020). Molecular and Functional Characterization of CaNAC035, an NAC Transcription Factor From Pepper (*Capsicum annuum* L.). Front. Plant Sci..

[18] Du X., Su M., Jiao Y., Xu S., Song J., Wang H., Li Q. (2022). A Transcription Factor SlNAC10 Gene of Suaeda liaotungensis Regulates Proline Synthesis and Enhances Salt and Drought Tolerance. Int. J. Mol. Sci..

[19] Shim J., Oh N., Chung P., Kim Y., Do Choi Y., Kim J. (2018). Overexpression of OsNAC14 Improves Drought Tolerance in Rice. Front. Plant Sci..

[20] Christianson J., Dennis E., Llewellyn D., Wilson I. (2010). ATAF NAC transcription factors: Regulators of plant stress signaling. Plant Signal Behav..

[21] Duval M., Hsieh T., Kim S., Thomas T. (2002). Molecular characterization of AtNAM: A member of the *Arabidopsis* NAC domain superfamily. Plant Mol. Biol..

[22] Olsen A., Ernst H., Leggio L., Skriver K. (2005). NAC transcription factors: Structurally distinct, functionally diverse. Trends Plant Sci..

[23] Zhao Y., Sun J., Xu P., Zhang R., Li L. (2014). Intron-mediated alternative splicing of wood-associated nac transcription factor1b regulates cell wall thickening during fiber development in Populus species. Plant Physiol..

[24] Sun Q., Huang J., Guo Y., Yang M., Guo Y., Li J., Zhang J., Xu W. (2020). A cotton NAC domain transcription factor, GhFSN5, negatively regulates secondary cell wall biosynthesis and anther development in transgenic *Arabidopsis*. Plant Physiol. Biochem..

[25] Shao H., Wang H., Tang X. (2015). NAC transcription factors in plant multiple abiotic stress responses: Progress and prospects. Front. Plant Sci..

[26] Singh S., Koyama H., Bhati K., Alok A. (2021). The biotechnological importance of the plant-specific NAC transcription factor family in crop improvement. J. Plant Res..

[27] Srivastava R., Kobayashi Y., Koyama H., Sahoo L. (2023). Cowpea NAC1/NAC2 transcription factors improve growth and tolerance to drought and heat in transgenic cowpea through combined activation of photosynthetic and antioxidant mechanisms. J. Integr. Plant Biol..

[28] Shan W., Kuang J., Lu W., Chen J. (2014). Banana fruit NAC transcription factor MaNAC 1 is a direct target of MaICE 1 and involved in cold stress through interacting with MaCBF 1. Plant Cell Environ..

[29] Wang Z., Chen Z., Wu Y., Mu M., Jiang J., Nie W., Zhao S., Cui G., Yin X. (2024). Genome-wide identification and characterization of NAC transcription factor family members in *Trifolium pratense* and expression analysis under lead stress. BMC Genom..

[30] Ling L., Li M., Chen N., Xie X., Han Z., Ren G., Yin Y., Jiang H. (2023). Genome-Wide Identification of NAC Gene Family and Expression Analysis under Abiotic Stresses in *Avena sativa*. Genes.

[31] Liu X., Zhou G., Chen S., Jia Z., Zhang S., He F., Ren M. (2024). Genome-wide analysis of the *Tritipyrum* NAC gene family and the response of *TtNAC477* in salt tolerance. BMC Plant Biol..

[32] Ming L., Zheng H., Jiang Q., Sun X., Yuan G., Qi J., Zhang H. (2018). GmNAC15 overexpression in hairy roots enhances salt tolerance in soybean. J. Integr. Agric..

[33] An J., Yao J., Xu R., You C., Wang X., Hao Y. (2018). An apple NAC transcription factor enhances salt stress tolerance by modulating the ethylene response. Physiol. Plant..

[34] Shinozaki K., Yamaguchi-Shinozaki K., Seki M. (2003). Regulatory network of gene expression in the drought and cold stress responses. Curr. Opin. Plant Biol..

[35] Wen B., Zhao X., Gong X., Zhao W., Sun M., Chen X., Li D., Li L., Xiao W. (2023). The NAC transcription factor *MdNAC4* positively regulates nitrogen deficiency-induced leaf senescence by enhancing ABA biosynthesis in apple. Mol. Hortic..

[36] Kurowska M., Daszkowska-Golec A. (2023). Molecular mechanisms of *SNAC1* (Stress-responsive NAC1) in conferring the abiotic stress tolerance. Plant Sci..

[37] Wang G., Liu Q., Shang X., Chen C., Xu N., Guan J., Meng Q. (2018). Overexpression of transcription factor SlNAC35 enhances the chilling tolerance of transgenic tomato. Biol. Plant..

[38] Zhao J., Wu Q., Wu H., Wang A., Wang X., Li C., Zhao H., Wu Q. (2022). FtNAC31, a *Tartary buckwheat* NAC transcription factor, enhances salt and drought tolerance in transgenic *Arabidopsis*. Plant Physiol. Biochem..

[39] Li M., Wu Z., Gu H., Cheng D., Guo X., Li L., Shi C., Xu G., Gu S., Abid M. (2021). AvNAC030, a NAC domain transcription factor, enhances salt stress tolerance in Kiwifruit. Int. J. Mol. Sci..

[40] Jin H., Huang F., Cheng H., Song H., Yu D. (2013). Overexpression of the *GmNAC2* Gene, an NAC Transcription Factor, Reduces Abiotic Stress Tolerance in Tobacco. Plant Mol. Biol. Rep..

[41] Han D., Han J., Xu T., Li X., Yao C., Li T., Sun X., Wang X., Yang G. (2021). Overexpression of *MbERF12*, an ERF gene from *Malus baccata* (L.) Borkh increases cold and salt tolerance in *Arabidopsis thaliana* associated with the ROS scavenging through ethylene signal transduction. In Vitro Cell. Dev. Biol. Plant.

[42] Hou X., Zhang H., Liu S., Wang X., Zhang Y., Meng Y., Luo D., Chen R. (2020). The NAC transcription factor CaNAC064 is a regulator of cold stress tolerance in peppers. Plant Sci..

[43] Hu X., Xie F., Liang W., Liang Y., Zhang Z., Zhao J., Hu G., Qin Y. (2022). HuNAC20 and HuNAC25, Two Novel NAC Genes from Pitaya, Confer Cold Tolerance in Transgenic *Arabidopsis*. Int. J. Mol. Sci..

[44] Moyano E., Martínez-Rivas F., Blanco-Portales R., Molina-Hidalgo F., Ric-Varas P., Matas-Arroyo A., Caballero J., Muñoz-Blanco J., Rodríguez-Franco A. (2018). Genome-wide analysis of the NAC transcription factor family and their expression during the development and ripening of the *Fragaria*× *ananassa* fruits. PLoS ONE.

[45] Dang X., Zhang B., Li C., Nagawa S. (2022). FvNST1b NAC protein induces secondary cell wall formation in strawberry. Int. J. Mol. Sci..

[46] Carrasco-Orellana C., Stappung Y., Mendez-Yanez A., Allan A., Espley R., Plunkett B., Moya-Leon M.A., Herrera R. (2018). Characterization of a ripening-related transcription factor *FcNAC1* from fragaria chiloensis fruit. Sci. Rep..

[47] Martín-Pizarro C., Vallarino J., Osorio S., Meco V., Urrutia M., Pillet J., Casanal A., Merchante C., Amaya I., Willmitzer L. (2021). The NAC transcription factor FaRIF controls fruit ripening in strawberry. Plant Cell..

[48] Zhang B., Dang X., Chen H., Li T., Zhu F., Nagawa S. (2023). Ectopic Expression of *FvVND4c* Promotes Secondary Cell Wall Thickening and Flavonoid Accumulation in *Fragaria vesca*. Int. J. Mol. Sci..

[49] Chen T., Wei W., Shan W., Kuang J., Chen J., Lu W., Yang Y. (2023). MaHDA6-MaNAC154 module regulates the transcription of cell wall modification genes during banana fruit ripening. Postharvest Biol. Technol..

[50] Yu X., Liu Y., Wang S., Tao Y., Wang Z., Shu Y., Peng H., Mijiti A., Wang Z., Zhang H. (2016). CarNAC4, a NAC-type chickpea transcription factor conferring enhanced drought and salt stress tolerances in *Arabidopsis*. Plant Cell Rep..

[51] Bi Y., Wang H., Yuan X., Yan Y., Li D., Song F. (2023). The NAC transcription factor ONAC083 negatively regulates rice immunity against Magnaporthe oryzae by directly activating transcription of the RING-H2 gene OsRFPH2-6. J. Integr. Plant Biol..

[52] Seo P., Kim S., Park C. (2008). Membrane-bound transcription factors in plants. Trends Plant Sci..

[53] Lee S., Lee H., Huh S., Paek K., Ha J., Park C. (2014). The Arabidopsis NAC transcription factor NTL4 participates in a positive feedback loop that induces programmed cell death under heat stress conditions. Plant Sci..

[54] Kim M., Park M., Seo P., Song J., Kim H., Park C. (2012). Controlled nuclear import of the transcription factor NTL6 reveals a cytoplasmic role of SnRK2. 8 in the drought-stress response. Biochem. J..

[55] Yang Z., Lu S., Wang M., Bi D., Sun L., Zhou S., Song Z., Liu J. (2014). A plasma membrane-tethered transcription factor, NAC 062/ANAC 062/NTL 6, mediates the unfolded protein response in *Arabidopsis*. Plant J..

[56] De Clercq I., Vermeirssen V., Van Aken O., Vandepoele K., Murcha M., Law S., Inzé A., Ng S., Ivanova A., Rombaut D. (2013). The membrane-bound NAC transcription factor ANAC013 functions in mitochondrial retrograde regulation of the oxidative stress response in *Arabidopsis*. Plant Cell..

[57] Yang Z., Wang M., Sun L., Lu S., Bi D., Sun L., Song Z., Zhang S.-S., Zhou S., Liu J. (2014). The membrane-associated transcription factor NAC089 controls ER-stress-induced programmed cell death in plants. PLoS Genet..

[58] Lu X., Zhang X., Duan H., Lian C., Liu C., Yin W., Xia X. (2018). Three stress-responsive NAC transcription factors from *Populus euphratica* differentially regulate salt and drought tolerance in transgenic plants. Physiol. Plant..

[59] Zhang S., Xu T., Ren Y., Song L., Liu Z., Kang X., Li Y. (2023). The NAC transcription factor family in *Eucommia ulmoides*: Genome-wide identification, characterization, and network analysis in relation to the rubber biosynthetic genes. Front. Plant Sci..

[60] Zhao X., Zhao J., Yang Q., Huang M., Song Y., Li M., Sui S., Liu D. (2023). Functional Characterization of the *CpNAC1* Promoter and Gene from *Chimonanthus praecox* in *Arabidopsis*. Int. J. Mol. Sci..

[61] Noctor G., Foyer C. (1998). Ascorbate and Glutathione: Keeping Active Oxygen Under Control. Annu. Rev. Plant Biol..

[62] Nadarajah K. (2020). ROS Homeostasis in Abiotic Stress Tolerance in Plants. Int. J. Mol. Sci..

[63] Matysik J., Alia, Bhalu B., Mohanty P. (2002). Molecular mechanisms of quenching of reactive oxygen species by proline under stress in plants. Curr. Sci..

[64] Bartels D., Sunkar R. (2005). Drought and salt tolerance in plants. CRC Crit. Rev. Plant Sci..

[65] Dai F., Zhou M., Zhang G. (2007). The change of chlorophyll fluorescence parameters in winter barley during recovery after freezing shock and as affected by cold acclimation and irradiance. Plant Physiol. Biochem..

[66] Tak H., Negi S., Ganapathi T. (2017). Banana NAC transcription factor MusaNAC042 is positively associated with drought and salinity tolerance. Protoplasma.

[67] Mao X., Chen S., Li A., Zhai C., Jing R. (2014). Novel NAC transcription factor *TaNAC67* confers enhanced multi-abiotic stress tolerances in *Arabidopsis*. PLoS ONE.

[68] Zhang J., Jia W., Yang J., Ismail A. (2006). Role of ABA in integrating plant responses to drought and salt stresses. Field Crops Res..

[69] Zhang X., Long Y., Huang J., Xia J. (2020). OsNAC45 is Involved in ABA Response and Salt Tolerance in Rice. Rice.

[70] Meng X., Liu S., Zhang C., He J., Ma D., Wang X., Dong T., Guo F., Cai J., Long T. (2022). The unique sweet potato NAC transcription factor IbNAC3 modulates combined salt and drought stresses. Plant Physiol..

[71] Liu J., Wang H., Su M., Li Q., Xu H., Song J., Li C., Li Q. (2023). A Transcription Factor SlNAC4 Gene of *Suaeda liaotungensis* Enhances Salt and Drought Tolerance through Regulating ABA Synthesis. Plants.

[72] Xu Z., Kim S., Dai J., Hyeon D., Kim D., Dong T., Park Y., Jin J., Joo S., Kim S. (2013). The *Arabidopsis* NAC Transcription Factor *ANAC096* Cooperates with bZIP-Type Transcription Factors in Dehydration and Osmotic Stress Responses. Plant Cell..

[73] Li M., Chen R., Jiang Q., Sun X., Zhang H., Hu Z. (2021). GmNAC06, a NAC domain transcription factor enhances salt stress tolerance in soybean. Plant Mol. Biol..

[74] Gao F., Xiong A., Peng R., Jin X., Xu J., Zhu B., Chen J., Yao Q. (2010). *OsNAC52*, a rice NAC transcription factor, potentially responds to ABA and confers drought tolerance in transgenic plants. Plant Cell Tiss. Organ. Cult..

[75] Narusaka Y., Nakashima K., Shinwari Z., Sakuma Y., Furihata T., Abe H., Narusaka M., Shinozaki K., Yamaguchi-Shinozaki K. (2003). Interaction between two cis-acting elements, ABRE and DRE, in ABA-dependent expression of *Arabidopsis* rd29A gene in response to dehydration and high-salinity stresses. Plant J..

[76] Liang T., Yu S., Pan Y., Wang J., Kay S. (2024). The interplay between the circadian clock and abiotic stress responses mediated by ABF3 and CCA1/LHY. Proc. Natl. Acad. Sci. USA.

[77] Yoshiba Y., Nanjo T., Miura S., Yamaguchi-Shinozaki K., Shinozaki K. (1999). Stress-responsive and developmental regulation of Δ1-pyrroline-5-carboxylate synthetase 1 (P5CS1) gene expression in *Arabidopsis thaliana*. Biochem. Biophys. Res. Commun..

[78] Soon F., Ng L., Zhou X., West G., Kovach A., Tan M., Suino-Powell K., He Y., Xu Y., Chalmers M.J. (2012). Molecular Mimicry Regulates ABA Signaling by SnRK2 Kinases and PP2C Phosphatases. Science.

[79] Thomashow M.F. (1999). Plant cold acclimation: Freezing tolerance genes and regulatory mechanisms. Annu. Rev. Plant Biol..

[80] Liu Y., Dang P., Liu L., He C. (2019). Cold acclimation by the CBF–COR pathway in a changing climate: Lessons from *Arabidopsis thaliana*. Plant Cell Rep..

[81] Zhou M., Shen C., Wu L., Tang K., Lin J. (2011). CBF-dependent signaling pathway: A key responder to low temperature stress in plants. Crit. Rev. Biotechnol..

[82] Haake V., Cook K., Riechmann J., Pineda O., Thomashow M., Zhang J. (2002). Transcription Factor CBF4 Is a Regulator of Drought Adaptation in *Arabidopsis*. Plant Physiol..

[83] Li J., Wang N., Xin H., Li S. (2013). Overexpression of VaCBF4, a Transcription Factor from *Vitis amurensis*, Improves Cold Tolerance Accompanying Increased Resistance to Drought and Salinity in Arabidopsis. Plant Mol. Biol. Rep..

[84] Mei C., Yang J., Mei Q., Jia D., Yan P., Feng B., Mamat A., Gong X.Q., Guan Q.M., Mao K. (2023). MdNAC104 positively regulates apple cold tolerance via CBF-dependent and CBF-independent pathways. Plant Biotechnol. J..

[85] Yong Y., Zhang Y., Lyu Y. (2019). A Stress-Responsive NAC Transcription Factor from Tiger Lily (LlNAC2) Interacts with LlDREB1 and LlZHFD4 and Enhances Various Abiotic Stress Tolerance in *Arabidopsis*. Int. J. Mol. Sci..

[86] Darwish O., Shahan R., Liu Z., Slovin J., Alkharouf N. (2015). Re-annotation of the woodland strawberry (*Fragaria vesca*) genome. BMC Genom..

[87] Han D., Zhang Z., Ni B., Ding H., Liu W., Li W., Chai L., Yang G. (2018). Isolation and functional analysis of *MxNAS3* involved in enhanced iron stress tolerance and abnormal flower in transgenic *Arabidopsis*. J. Plant Interact..

[88] Li X., Liang X., Li W., Yao A., Liu W., Wang Y., Yang G., Han D. (2022). Isolation and functional analysis of *MbCBF2*, a *Malus baccata* (L.) Borkh CBF transcription factor gene, with functions in tolerance to cold and salt stress in transgenic *Arabidopsis thaliana*. Int. J. Mol. Sci..

[89] Han D., Shi Y., Wang B., Liu W., Yu Z., Lv B., Yang G. (2015). Isolation and preliminary functional analysis of *MxCS2*: A gene encoding a citrate synthase in *Malus xiaojinensis*. Plant Mol. Biol. Rep..

[90] Zhang L., Zhu L., Xu Y., Lv L., Li X., Li W., Liu W., Ma F., Li M., Han D. (2023). Genome-wide identiffcation and function analysis of the sucrose phosphate synthase *MdSPS* gene family in apple. J. Integr. Agric..

[91] Kumar S., Stecher G., Tamura K. (2016). MEGA7: Molecular evolutionary genetics analysis version 7.0 for bigger datasets. Mol. Biol. Evol..

[92] Li Y., Zhong J., Huang P., Shao B., Li W., Liu W., Wang Y., Xie L., Han M., Han D. (2022). Overexpression of *MxFRO6*, a FRO gene from *Malus xiaojinensis*, increases iron and salt tolerance in *Arabidopsis thaliana*. In Vitro Cell. Dev. Biol. Plant.

[93] Han D., Wang L., Wang Y., Yang G., Gao C., Yu Z., Li T., Zhang X., Ma L., Xu X. (2013). Overexpression of *Malus xiaojinensis* CS1 gene in tobacco affects plant development and increases iron stress tolerance. Sci. Hortic..

[94] Han D., Hou Y., Ding H., Zhou Z., Li H., Yang G. (2018). Isolation and preliminary functional analysis of *MbWRKY4* gene involved in salt tolerance in transgenic tobacco. Int. J. Agric. Biol..

[95] Han J., Li X., Li W., Yang Q., Li Z., Cheng Z., Lv L., Zhang L., Han D. (2023). Isolation and preliminary functional analysis of FvICE1, involved in cold and drought tolerance in *Fragaria vesca* through overexpression and CRISPR/Cas9 technologies. Plant Physiol. Biochem..

[96] Ren C., Luo G., Li X., Yao A., Liu W., Zhang L., Wang Y., Li W., Han D. (2023). *MxFRO4* confers iron and salt tolerance through up-regulating antioxidant capacity associated with the ROS scavenging. J. Plant Physiol..

[97] Zhang J., Kirkham M. (1994). Drought-stress-induced changes in activities of superoxide dismutase, catalase, and peroxidase in wheat species. Plant Cell Physiol..

[98] Kumar D., Yusuf M., Singh P., Sardar M., Sarin N. (2014). Histochemical detection of superoxide and H_2_O_2_ accumulation in *Brassica juncea* seedlings. Bio-Protocol.

[99] Liu W., Liang X., Cai W., Wang H., Liu X., Cheng L., Song P., Luo G., Han D. (2022). Isolation and functional analysis of VvWRKY28, a *Vitis Vinifera* WRKY transcription factor gene, with functions in tolerance to cold and salt stress in transgenic *Arabidopsis thaliana*. Int. J. Mol. Sci..

[100] Li W., Zhong J., Zhang L., Wang Y., Song P., Liu W., Li X., Han D. (2022). Overexpression of a *Fragaria vesca* MYB transcription factor gene (FvMYB82) increases salt and cold tolerance in *Arabidopsis thaliana*. Int. J. Mol. Sci..

[101] Malnoy M., Jin Q., Borejsza-Wysocka E., He S., Aldwinckle H. (2007). Overexpression of the Apple *MpNPR1* Gene Confers Increased Disease Resistance in *Malus × domestica*. Mol. Plant. Microbe Interact..

