# Supplementary material for: Overexpression of a Fragaria vesca NAM, ATAF, and CUC (NAC) Transcription Factor Gene (FvNAC29) Increases Salt and Cold Tolerance in Arabidopsis thaliana
Source: Int J Mol Sci. 2024 Apr 6;25(7):4088. doi: 10.3390/ijms25074088 (PMC11012600; doi:10.3390/ijms25074088)
Supplement: Supplementary file 1 [file ijms-25-04088-s001.zip › ijms-2929691-supplementary.pdf]

Supplementary Materials

Table S1. List of primers used in this study.

Primer Name	PrimerSequence (5′→3′)	Purpose
FvActin-F	GCGACAATGGAACTGGAATGG	qPCR
FvActin-R	GACAATTTCCCGTTCAGCAGTG	qPCR
FvNAC29-qF	AAGCCAATAGTAGCTCTGGACT	qPCR
FvNAC29-qR	GTTGTTGTACGCTGGATTCAC	qPCR
FvNAC29-F	ATGGAAGCCAATAGTAGCTC	full-length cDNA of *FvNAC29*
FvNAC29-R	TTAGTGGTTCAAACCATTCATG	full-length cDNA of *FvNAC29*
HR-F	AGAACACGGGGGACGAGCTCATGGAAGCCAATAGTAGCTC	PCR for homologous recombination
HR-R	ACCATGGTGTCGACTCTAGATTAGTGGTTCAAACCATTCATG	PCR for homologous recombination
FvNAC29-slF	GGATCCCCTAGGCAAAAGAGTGAAAG	For subcellular localization
FvNAC29-slR	TCACTTATGAAATAGCCATGAAACC	For subcellular localization
